# The Biochemical, Microbiological, Antioxidant and Sensory Characterization of Fermented Skimmed Milk Drinks Supplemented with Probiotics *Lacticaseibacillus casei* and *Lacticaseibacillus rhamnosus*

**DOI:** 10.3390/microorganisms11102523

**Published:** 2023-10-09

**Authors:** Iqra Shabbir, Fahad Al-Asmari, Hafiza Saima, Muhammad Tahir Nadeem, Saadia Ambreen, Ladislaus Manaku Kasankala, Muhammad Zubair Khalid, Muhammad Abdul Rahim, Fatih Özogul, Elena Bartkiene, João Miguel Rocha

**Affiliations:** 1 Department of Food Science, Faculty of Life Sciences, Government College University, Faisalabad 38000, Pakistan; iqrashabbir1304@gmail.com (I.S.); drhafizasaima@gcuf.edu.pk (H.S.); or mt.nadeem@gcuf.edu.pk (M.T.N.); zubairkhalid730@gmail.com (M.Z.K.); 2 Department of Food and Nutrition Sciences, College of Agricultural and Food Sciences, King Faisal University, Al-Ahsa 31982, Saudi Arabia; falasmari@kfu.edu.sa; 3 University Institute of Food Science & Technology, The University of Lahore, Lahore 54590, Pakistan; saadia.ambreen@uifst.uol.edu.pk; 4 Department of Food Science and Nutrition, Tanzania and Nutrition Centre, Dar es Salaam 11101, Tanzania; ladislaus.kasankala@tfnc.go.tz; 5 Department of Seafood Processing Technology, Faculty of Fisheries, Cukurova University, Balcali, Adana 01330, Turkey; fozogul@cu.edu.tr; 6 Biotechnology Research and Application Center, Cukurova University, Balcali, Adana 01330, Turkey; 7 Department of Food Safety and Quality, Faculty of Veterinary, Lithuanian University of Health Sciences, Tilzes Str. 18, LT-47181 Kaunas, Lithuania; elena.bartkiene@lsmu.lt; 8 Faculty of Animal Sciences, Institute of Animal Rearing Technologies, Lithuanian University of Health Sciences, Tilzes Str. 18, LT-47181 Kaunas, Lithuania; 9 Universidade Católica Portuguesa, CBQF—Centro de Biotecnologia e Química Fina—Laboratório Associado, Escola Superior de Biotecnologia, Rua Diogo Botelho 1327, 4169-005 Porto, Portugal; 10 LEPABE—Laboratory for Process Engineering, Environment, Biotechnology and Energy, Faculty of Engineering, University of Porto, Rua Dr. Roberto Frias, s/n, 4200-465 Porto, Portugal; 11 ALiCE—Associate Laboratory in Chemical Engineering, Faculty of Engineering, University of Porto, Rua Dr. Roberto Frias, s/n, 4200-465 Porto, Portugal

**Keywords:** lactic acid bacteria (LAB), *Lacticaseibacillus casei*, *Lacticaseibacillus rhamnosus*, Yakult-like fermented skimmed milk drink, chemical composition, sensory analysis, physicochemical, antioxidant, microbiological quality

## Abstract

A variety of foods fermented with lactic acid bacteria (LAB) serve as dietary staples in many countries. The incorporation of health-promoting probiotics into fermented milk products can have profound effects on human health. Considering the health benefits of Yakult, the current study was undertaken to develop an enriched Yakult-like fermented skimmed milk drink by the addition of two probiotic strains, namely *Lacticaseibacillus casei* (Lc) and *Lacticaseibacillus rhamnosus* (Lr). The prepared drinks were compared in terms of various parameters, including their physicochemical properties, proximate chemical composition, mineral estimation, microbial viable count, antioxidant activity, and sensory evaluation. Each strain was employed at five different concentrations, including 1% (T_1_), 1.5% (T_2_), 2% (T_3_), 2.5% (T_4_), and 3% (T_5_). The prepared Yakult samples were stored at 4 °C and analyzed on days 0, 7, 14, 21, and 28 to evaluate biochemical changes. The findings revealed that the concentration of the starter culture had a significant (*p* ≤ 0.05) impact on the pH value and moisture and protein contents, but had no marked impact on the fat or ash content of the developed product. With the Lc strain, Yakult’s moisture content ranged from 84.25 ± 0.09 to 85.65 ± 0.13%, whereas with the Lr strain, it was from 84.24 ± 0.08 to 88.75 ± 0.13%. Protein levels reached their highest values with T_5_ (3% concentration). The acidity of all treatments increased significantly due to fermentation and, subsequently, pH showed a downward trend (*p* ≤ 0.05). The total soluble solids (TSS) content decreased during storage with Lc as compared to Lr, but the presence of carbohydrates had no appreciable impact. The drink with Lc exhibited a more uniform texture and smaller pore size than Yakult with Lr. Except for the iron values, which showed an increasing trend, the contents of other minerals decreased in increasing order of the added probiotic concentration used: 1% (T_1_), 1.5% (T_2_), 2% (T_3_), 2.5% (T_4_), and 3% (T_5_). The highest lactobacilli viable count of 8.69 ± 0.43 colony-forming units (CFU)/mL was observed with the T_1_ Lr-containing drink at the end of the storage period. Regarding the storage stability of the drink, the highest value for DPPH (88.75 ± 0.13%) was found with the T_1_ Lc drink on day 15, while the highest values for FRAP (4.86 ± 2.80 mmol Fe^2+^/L), TPC (5.97 ± 0.29 mg _GAE_/mL), and TFC (3.59 ± 0.17 mg _GAE_/mL) were found with the T_5_ Lr drink on day 28 of storage. However, the maximum value for ABTS (3.59 ± 0.17%) was noted with the T_5_ Lr drink on the first day of storage. The results of this study prove that Lc and Lr can be used in dairy-based fermented products and stored at refrigerated temperatures.

## 1. Introduction

Probiotics are defined as live microorganisms that provide health benefits to the host when consumed in adequate amounts. Probiotic-containing products have long been studied and praised for their positive effects on gastrointestinal health [1,2]. Yakult and other non-alcoholic fermented milk-based drinks are among the most suitable products for the addition of probiotic microorganisms [3]. Probiotics can prevent and treat illnesses like lactose intolerance, combustible bowel disease, gastrointestinal disorders, rotavirus diarrhea, food allergies, and colonic disorders; therefore, they should be made available as dietary supplements [4]. Probiotics should play a bigger part in terms of their sustainable growth in order to promote wellness. A number of genera of bacteria and yeast have been used as probiotics in food products for multiple purposes, including *Lacticaseibacillus, Leuconostoc, Pediococcus*, *Bifidobacterium*, and *Enterococcus*, but the main species believed to have probiotic characteristics are *Lacticaseibacillus acidophilus*, *Bifidobacterium* spp., and *Lacticaseibacillus casei* (Lc). Yakult is produced using a strain of lactic acid bacteria (LAB), namely Lc. The small intestine can become colonized by gastro-resistant Lc, which also produce antimicrobial substances that boost macrophage activity and proliferation. Yakult contains around 1.4 g of milk protein per 100 mL and is categorized by the codex standard (Rome, Italy) as a fermented milk drink. Owing to its human health benefits, Yakult is available in 15 countries, including some European countries [5,6,7]. On the other hand, the *Lacticaseibacillus rhamnosus* (Lr) strain has been used in various products, such as yogurt, cheese, and other products, to boost the probiotic content of the host. In many studies, its health benefits are well documented and include the prevention and treatment of metabolic disorders, respiratory tract infections, oxidative stress, and allergic reactions. Additional benefits of Lr include the prevention of spoilage, increased food safety, and increased nutritional value by providing vitamins [8,9]. Probiotic starter cultures containing the most commonly documented probiotic strains, *Lacticaseibacillus casei* (Lc) and *Lacticaseibacillus rhamnosus* (Lr), were used to produce enriched Yakult-like fermented skimmed milk drinks. This study aimed to characterize the chemical composition and sensory, physicochemical, antioxidant, and microbiological qualities of probiotic Yakult-like fermented skimmed milk drinks.

## 2. Materials and Methods

The skimmed milk powder used for manufacturing the Yakult-like fermented skimmed milk was Skimmillac, purchased from Millac Foods Pvt Ltd. (Karachi, Sindh, Pakistan). Other ingredients, including glucose, sugar, and natural flavor (citrus), were purchased from the local supermarket in Faisalabad, Punjab, Pakistan. All chemicals were purchased from Merck (Merck KGaA, Darmstadt, Germany) and Sigma-Aldrich (Sigma-Aldrich, Tokyo, Japan). The probiotic strains *Lacticaseibacillus casei* (Lc) and *Lacticaseibacillus rhamnosus* (Lr) were isolated from lactase. Lc and Lr were cultivated on de Man, Rogosa and Sharpe (MRS) broth (Oxoid, Ltd., Basingstoke, Hampshire, UK) and incubated for 15 to 18 h at 37 °C anaerobically, and the activated culture was again inoculated into MRS broth at 37 °C for 18 h. The microbial cultures were kept in broth medium in a cold store (4 °C) with tight caps until they were needed, in order to keep the cultures fresh for later use.

### 2.1. Fermented Skimmed Milk Drink Manufacturing Process and Experimental Design

The skimmed milk powder (500 g) was added to water and heated to 90 °C for 60 min in a sterile stainless-steel container (local market, Punjab, Pakistan). A total of 10 g of sugar (sucrose and dextrose) was mixed with sterilized water and added to the milk under aseptic conditions. The milk solution was aseptically inoculated with either the *Lacticaseibacillus casei* strain or the *Lacticaseibacillus rhamnosus* strain at five different concentrations, as described in Table 1. The fermentation was allowed to proceed at a controlled temperature of 37 °C in an incubator until the number of Lc and Lr strains reached a cell density of 8 log (CFU/mL), determined using a spectrophotometer (Analytik Jena AG Specord 200 Plus, Jena, Germany), and the sweet milk solution became transformed into a yoghurt-like a curd, known as a cultural base. This step generally took 6–7 days. After the fermentation, the Yakult-like fermented skimmed milks containing Lc and the comparison strain Lr were cooled at a temperature of 4 °C and stored for 28 days. Sampling was carried out at days 0, 7, 14, 21, and 28 of storage.

### 2.2. Physicochemical and Proximate Composition Analysis of Fermented Skimmed Milk Drinks

Samples of the Yakult-like fermented skimmed milk drinks were evaluated for their physiochemical and proximate compositions on days 0, 7, 14, 21, and 28 of storage using AOAC analytical methods [10]. The moisture content of the fermented skimmed milk drinks was calculated using the prescribed method number 934.06. All samples were placed in a hot air oven (Memmert, Germany) at 105 °C for 60 min. The Kjeldhal method was used to determine the protein content via standard method number 981.10. The Gerber method (method number 2000.18) was used to determine the fat content, using a butyrometer. The amount of ash in the fermented skimmed milk drinks was determined at 550–650 °C for 5–6 h in a muffle furnace (method number 930.30). Before being charred, the sample was weighed and heated until no flame was visible. The lactose (carbohydrate) content was determined using the titrimetric method with Fehling A solution (69.3 g CuSO_4_ dissolved in water and made up to a volume of 1 L) and Fehling B solution (100 g NaOH and 365 g sodium tartrate salt dissolved in water and made up to a volume of 1 L) [11]. The total titratable acidity (TTA) was measured using phenolphthalein indicator and the total soluble solids (TSS) were calculated according to AOAC method numbers 947.05 and 961.07, respectively. The pH of the fermented skimmed milk drinks was determined using a pH meter (ST2100-F, Melbourne, Australia) calibrated with pH 4 and 7 buffers according to the AOAC [10]. A Brookfield laboratory viscometer (Lab Viscometer, Barcelona, Spain) was used to measure the viscosity of the Yakult-like fermented skimmed milk drinks, which is one of the prerequisites for a probiotic milk, at a constant speed of 100 rpm and constant temperature using spindle number S–63.

### 2.3. Mineral Estimation of Fermented Skimmed Milk Drinks

The amounts of minerals in the prepared drinks were determined on days 0, 7, 14, 21, and 28 using a spectrophotometer (Analytik Jena AG-Specord 200 Plus, Jena, Germany). Until a clear solution could be seen, 50 mL of milk sample was digested on a hotplate with 5 mL of 65% HNO_3_ and 2 mL of 35% H_2_O_2_. Following filtering, the samples were diluted with distilled water to a volume of 50 mL before being subjected to additional analysis with a flame photometer (Model 410, Cambridge, UK) and spectrophotometer.

### 2.4. Microbial Viable Counts of Lacticaseibacillus in Fermented Skimmed Milk Drinks

The viabilities of Lc and Lr were evaluated during refrigeration at 4 °C for 28 days. Decimal dilutions were inoculated in sterilized Petri dishes with MRS agar and incubated for 48–72 h at 37 °C in anaerobiosis with an atmosphere of 5% CO_2_. After incubation, the colonies were counted and expressed as log CFU/mL [12]. The analysis was performed on days 0, 7, 14, 21, and 28 of storage.

### 2.5. Determination of the Antioxidant Activity of Fermented Skimmed Milk Drinks

A 20 mL sample of extraction solution [methanol/water (70:30, *v*/*v*)] was combined with 2 mL of the Yakult-like fermented skimmed milk samples and stirred for 4 h at 20 °C in an incubator (Memmert^®^ IN55, Memmert, Schwabach, Germany). The suspension was centrifuged and run through filter paper. The 2, 2-diphenyl-1-picrylhydrazyl (DPPH) free radical scavenging activity assay, ferric-reducing antioxidant power (FRAP) assay, 2, 29-azinobis (3-ethylene benzothiazoline) 6-sulphonic acid (ABTS) assay, total phenolic content (TPC) assay, and total flavonoid content (TFC) assay were used to measure the antioxidant activity of the suspension [13]. Assessment was made on days 0, 7, 14, 21, and 28 of storage.

#### 2.5.1. DPPH Assay

The stable DPPH radical, which has maximum absorbance at a wavelength of 517 nm, was used to calculate the free radical scavenging capacity of the fermented skimmed milk samples, conducted as per the method outlined in [14]. The assay was based on the capturing of DPPH radical by antioxidants. As a result, the absorbance measured at 517 nm was noted to be reduced. Briefly, 1 mL of fermented skimmed milk was mixed with 1 mL of fresh solution of DPPH (60 µM) in methanol and homogenized. The mixture was incubated in the dark for 45 min before measuring the absorbance at 517 nm with a spectrophotometer. Correspondingly, for the blank reading, the fermented skimmed milk sample was replaced by distilled water (1 mL). The DPPH radical scavenging activity was calculated as follows (Equation (1)):

DPPH-RSA (%) = (Absorbance for blank − Absorbance for the sample)/Absorbance for blank] × 100(1)

#### 2.5.2. FRAP Assay

The FRAP assay converts the ferric ion complex, Fe^3+^-TPTZ [iron(III)-2,4,6-tripyridyl-S-triazine], into a ferrous ion complex (Fe^2+^-TPTZ), which is violet in color. The change in absorbance at a wavelength of 700 nm was used to calculate the fermented skimmed milks’ antioxidant activity with a spectrophotometer. In this assay, TPZT solution, 20 mM ferric solution, and 0.3 M acetate buffer at pH 3.6 were used in a ratio of 10:1:1 (*v*/*v*), respectively, to prepare the final functional FRAP reagent. The number of milligrams of Trolox equivalent (TE) per gram was used to measure the capacity of the extracts to reduce iron (III). The data were expressed in mg TE/g units (milligram equivalents of Trolox per gram) [15,16].

#### 2.5.3. ABTS Assay

Analysis was performed using the technique reported by Ozgen [17]. For that purpose, 5 mL of ABTS stock solution (7 mM) was mixed with 2.5 mM K_2_SO_4_ (88 µL) and the mixture was placed in the dark for 12–15 h at around 25 °C prior to further use in the analysis. Later, the previously prepared ABTS^•+^ solution was mixed with phosphate-buffered saline (5 mM) at pH 7.4 until the absorbance was obtained by spectrophotometry at 734 nm. Afterwards, a mixture of diluted ABTS^•+^ solution (1mL) and the fermented skimmed milk sample (20 µL) was prepared and placed aside for 6 min at 30 °C. Thereafter, the mixture was placed in a spectrophotometer and the obtained absorbance at 734 nm was noted and plotted as a function of the reference antioxidant (Trolox, Sigma-Aldrich, Tokyo, Japan). Correspondingly, for the blank reading, distilled water (20 µL) was used in place of the Yakult-like sample. The ABTS-radical scavenging activity was computed using Equation (2):

RSA (%) = [(Absorbance of blank − Absorbance of sample)/Absorbance of blank] × 10(2)

#### 2.5.4. TPC

By using this quantitative method, also referred to as the Folin–Ciocalteu (FC) reducing assay, the total phenolic content was determined. Oxidation of phenolic compounds reduces the FC reagent, producing a blue endpoint, and it is then measured at 765 nm using gallic acid as a standard with a spectrophotometer [16,18]. The results were expressed as mg_GAE_/mL.

#### 2.5.5. Total Flavonoid Content (TFC)

The total flavonoid content of the product was quantified using a spectrophotometer. A mixture of 100 mL of ethanol, 70 mL of AlCl_3_, and 70 mL of NaNO_3_ was combined with 1 L of the sample extract. This was left for 6 min after being vigorously shaken; then, 50 mL NaOH was added and diluted with 2500 mL distilled water. The absorbance was measured at 440 nm after 10 min [13,16,19]. Each determination was made three times and expressed as mg of quercetin-equivalent (QE) per mL.

### 2.6. Sensory Evaluation of Fermented Skimmed Milk Drinks

The sensory attributes were evaluated directly after preparation of the Yakult-like fermented skimmed milks. Consumers use sensory evaluation to rate the sensory qualities of foods and beverages, and it also gives manufacturers information relevant to product development and marketing tactics. The prepared drinks were evaluated on a 9-point hedonic scale (1–9 and the sensory scores for color, flavor, taste, texture, and overall acceptability were categorized as “1 = extremely disliked” to “9 = extremely liked.” Sensory evaluation of the fermented skimmed milk drinks was performed by 15 trained evaluators, selected and trained according to a standardized method. Reference materials for quantitative and qualitative calibration of the panel were obtained through familiarization with scale definitions [20,21].

### 2.7. Statistical Analysis

Each analysis was performed in triplicate. The results were calculated using Statistix version 8.1. The data were presented in the form of a mean, standard deviation (SD), and 95% confidence interval. Two-way analysis of variance (ANOVA) was used to further evaluate the normally distributed data at *p* ≤ 0.05 to investigate the fermented skimmed milks’ physicochemical, mineral, antioxidant, and sensory qualities, as well as mineral estimation.

## 3. Results and Discussion

### 3.1. Physicochemical and Proximate Composition of Fermented Skimmed Milk Drinks

Table 2 and Table 3 show the proximate composition of the prepared drinks (all treatments) using the two probiotic strains, *Lacticaseibacillus casei* (Lc) and *Lacticaseibacillus rhamnosus* (Lr). The findings demonstrated that the moisture, fat, and ash contents of the samples were non-significantly affected by the increasing microbial culture concentration. In comparison to the Yakult-like Lr drink (Yakult-like fermented skimmed milk with Lr), which had a slightly higher moisture content (88.65%) at T_1_ on day 0, the Yakult-like Lc drink (Yakult-like fermented skimmed milk with Lc) had a moisture content of 85.22% at T_1_ on day 0 and 84.77% at T_1_ on day 28. The fat content was higher (0.33 ± 0.21%) at T_1_ with the Yakult-like Lc drink than with the Yakult-like Lr drink (0.29 ± 0.21%) on day 0. The protein content of the Yakult-like Lc drink was higher (4.19 ± 0.24%) than that of the Yakult-like Lr drink (4.09 ± 0.30%) on day 28. In contrast, the Yakult-like Lr drink had the lowest ash content (0.49 ± 0.11%) on day 0 and the highest (0.53 ± 0.09%) on day 28, whereas the Yakult-like Lc drink had the lowest ash content (0.42 ± 0.01%) on day 0, which further decreased with increased concentration. The ash content of milk ranges from 8.2 to 6.8%, owing primarily to the amount of calcium (133.25 mg), magnesium (11.35 mg), and iron (0.27 mg) per 100 g of milk. The maximum amount of ash allowed in Yakult is 0.30. However, the carbohydrate content of the Yakult-like Lc drinks was discovered to be higher (8.85–10.69%) as compared to the Yakult-like Lr drinks.

The results of proximate analysis of the Yakult-like fermented skim milks (all treatments) using the two probiotic strains, Lc and Lr, correlate with the findings by Pato et al. [22], who reported that fermentation did not affect the moisture, fat, and ash contents of fermented milk. During fermentation, the moisture and ash contents in the media remained relatively constant due to the constant water and mineral requirements of lactic acid bacteria (LAB). The increase in ash content might have been due to the loss of moisture and breakdown of fats and carbohydrate. Protein content is an important factor that affects the quality of acid coagulation of protein gel products. The enhancement in protein content of the fermented milks with probiotic strains might have been due to some anabolic processes that lead to polymer build-up or microbial cell proliferation [23]. Hu et al. [24] reported an increase in protein levels in yogurt during fermentation from 3 to 15 h. García-Cano et al. [25] stated that lipolysis was significant in the fermentative processes of L. strains because the hydrolysis of triacylglycerols and proteins in milk produce favorable compounds. The longer the fermentation time period, the greater the breakdown of triacylglycerol into fatty acids and glycerol. The lower carbohydrate levels with longer fermentation times may have been due to the lactose and sucrose sugars in the fermentation medium being converted to lactic acid [26].

Table 4 and Table 5 show the total soluble solids (TSS), total titratable acidity (TTA), pH, and viscosity of the prepared Yakult-like drinks. The TSS content of the Yakult-like Lc drink (T_1_) was 10.58 ± 0.52% on day 0, which increased to 13.87 ± 0.69% on day 28. However, for the Yakult-like Lr drink, the TSS content was highest at T_1_ (11.35 ± 0.56%) and at its minimum at T_5_ (10.21 ± 0.55%). The maximum TTA value was at T_1_ (1.32 ± 0.05%) on day 28 for the Yakult-like Lc drink and was lower for the Yakult-like Lr drink at T_1_ (0.29–0.01%) on day 28. The pH value of the Yakult-like Lc drink was highest at T_1_ (4.49%) on day 0 and decreased throughout the course of the 28 days. The lowest pH value was observed at T_5_ (3.76%) on day 28 for the Yakult-like Lr drink. The minimum viscosity was observed at T_1_ (10.64%) for the Yakult-like Lc drink on day 0, which was slightly lower than that of the Yakult-like Lr drink at T_1_ (11.41%) due to the reduced ability of proteins to bind to water during storage, as well as the decreased number of probiotic bacteria hydrolyzing proteins and increasing the viscosity during storage. The findings showed that TSS, pH, and viscosity were significantly affected by the storage interval and interactions (*p* ≤ 0.05). For both strains, the probiotic culture concentration had a substantial effect on the characteristics of Yakult. The results of this study are comparable to those of previous investigations that found that the pH of yogurt fermented for 21 days was reduced from 4.51 to 3.95 [27] and the pH of Brazilian kefir fermented with LAB for 28 days at a refrigerated temperature was reduced from 4.75 to 4.32 [28]. As a result, it is important to highlight that the pH of probiotic fermented milk is affected by the storage time. A decrease in the pH of fermented milk corresponds concomitantly with lowered sucrose and lactose levels during cold storage. The decrease in pH value for the probiotic Yakult-like Lc and Yakult-like Lr drinks was due to the metabolism of lactose and sucrose into lactic acid. Similarly, regarding the study by Li et al. [12] and their results, in terms of titratable acidity after 28 days of storage, they noticed a significant increase in the acidity levels of fermented soymilk samples. The predominant acid in milk beverage samples is determined by TTA. Based on the results shown, TTA values were higher or lower than 0.19%. Due to the increased lactic acid production by LAB, TTA typically rises with storage. The more acid is formed, the more lactic acid is produced by LAB [29]. The acidity of fermented milk is due to LAB breaking down lactose and eventually other sugars. Acid–base titration, which estimates the total acid concentration, is used to determine the total acid in food. A previous study showed that the fermentation process, which results from the accumulation of acids from LAB, lowers the pH level. Protein is used to promote the growth of LAB, which use lactose as a carbon source and produce lactic acid as a metabolite, which lowers the pH level [30].

### 3.2. Microbial Viable Counts of L. casei and L. rhamnosus in Fermented Skimmed Milk Drinks

Table 6 shows the Lc and Lr viable counts in the fermented skimmed milk samples at various treatments under study during storage for 28 days at 4 °C. The results indicated that at T_1_ [5.74 ± 0.28 log (CFU/mL)], the minimum viable count was observed at day 0. At T_1_ [7.34 ± 0.36 log (CFU/mL)], the microbial viable count was found to be highest in the Yakult-like Lr drink on day 0. Moreover, an increasing trend was observed and the maximum viable count was observed at T_5_ (8.42 ± 0.42 log CFU/mL in Lr) on day 28. In the Yakult-like Lc drink, the microbial viable count was found to be lower than in the Yakult-like Lr drink at T_5_ (6.58 ± 0.31 log CFU/mL) on day 28. The increase in viable count was accompanied by a decrease in pH and increase in TTA as a result of the microbial activity. Observing the results from Table 6, it can be seen that the *lacticaseibacillus* viable counts were always higher in the Yakult-like fermented skimmed milks with Lr in comparison to the Yakult-like fermented skimmed milks with Lc. This finding shows that, eventually, fermentation with Lr can be a good option for the preparation of fermented skimmed milk drinks.

The slow growth of LAB is due to the low lactose content of fermented milk and low storage temperatures. As a result, the total numbers of LAB and LAB activity are reduced during cold storage [31]. In contrast, the total amount of LAB in Brazilian kefir remained constant during cold storage [28]. A study has shown that fermented milk products or food products need to contain at least 10^8^ log CFU/mL probiotic strains [32], which is approximately the recommended daily intake of viable probiotics in fermented milk. As expected, our data showed that the fermented skimmed milks contained significantly more LAB during storage when the culture concentration was increased. When the culture concentrations were greater than 1%, the amount of LAB in all treatments increased [33]. The most important factor in preserving LAB viability in fermented beverages and extending their shelf-life is refrigeration [34,35]. Higher concentrations of probiotics contributed to the significant (*p* ≤ 0.05) increase in viable numbers of these strains. Darwaish et al. [36] discovered that the growth pattern of the *Lactobacillus acidophilus* strain was substantially enhanced in bio-yogurt samples for up to 14 days, after which the viable count decreased, although it remained adequate. This decrease could have been because of a nutrient deficiency in the bio-yogurt samples.

### 3.3. Antioxidant Activity of Fermented Skimmed Milk Drinks

Table 7 and Table 8 show the total phenolic content (TPC), total flavonoid content (TFC), 2-diphenyl-1-picrylhydrazyl (DPPH) free radical scavenging activity, ferric-reducing antioxidant power (FRAP), and 2, 29-azinobis (3-ethylene benzothiazoline) 6-sulphonic acid (ABTS) radical scavenging activity of the Yakult-like fermented skimmed milks containing *Lacticaseibacillus casei* (Lc) and *Lacticaseibacillus rhamnosus* (Lr) strains. For the Yakult-like Lc drink, the lowest mean value of DPPH was at T_5_ (84.24 ± 0.14%) on day 28, but for the Yakult-like Lr drink, the lowest value was at T_1_ (30.61 ± 1.53%), which occurred on day 0. The highest mean value was observed at T_1_ (88.65 ± 0.14%) on day 0 for the Yakult-like Lc drink. The lowest value of FRAP was at T_1_ (0.23 ± 0.01 mmol Fe^2+^/L), while the maximum value was observed at T_5_ (0.71 ± 0.03 mmol Fe^2+^/L) on day 0. For the Yakult-like Lc drink and Yakult-like Lr drink, the maximum value was observed on day 28 at T_5_ (4.53 ± 2.67%). The highest mean value of ABTS was at T_5_ (87.69 ± 4.15%) on day 0 for the Yakult-like Lr drink and (84.69 ± 4.15%) for the Yakult-like Lc drink. On day 28, the lowest value of ABTS was at T_1_ (31.43 ± 1.57%) for the Yakult-like Lc drink, while it was (48.57 ± 2.42%) for the Yakult-like Lr drink. The minimum value of TPC was observed at T_1_ (3.38 ± 0.16%) on day 0 for the Yakult-like Lr drink and the maximum value occurred at T_1_ [4.61 ± 0.23 mg gallic-acid-equivalent (GAE)/g] for the Yakult-like Lc drink. On day 28, the TFC content was (0.37 ± 0.01 mg _QE_/mL) in the Yakult-like Lc drink, while for the Yakult-like Lr drink, the maximum vale of TFC was at T_5_ (3.51 ± 0.17 mg _QE_/mL). These results support earlier research that discovered fermented plant beverages with different *Lacticaseibacillus* strains had high antioxidant activity [37,38]. During fermentation, LAB cultures are responsible for increasing the total phenolic content due to the increased antioxidant activity [39]. However, fermentation increases the overall antioxidant capacity because the microbiota has proteolytic activity, creating protein fractions [40]. The peptides produced by proteolysis act as electron donors in fermented milk and react with free radicals to form more stable products. Inhibiting free radicals as a metabolite, lactic acid can also function as a chelating agent [41]. The increase in TPC and TFC was caused by fermentation and the concomitant microbial hydrolysis, thus increasing the amounts of phenolic and flavonoid compounds. Additionally, owing to possible structural disintegration of the cell wall, several antioxidant compounds are released or produced [42].

### 3.4. Mineral Estimation of Fermented Skimmed Milk Drinks

Table 9 and Table 10 show the mineral content of the Yakult-like fermented skimmed milk samples containing *Lacticaseibacillus casei* (Lc) and *Lacticaseibacillus rhamnosus* (Lr). The mineral content showed a slight decrease with increased concentration of probiotic LAB. The calcium content was higher at T_1_ (9.64 ± 0.48 mg/100 g) and lower at T_5_ (9.48 ± 0.47 mg/100 g) at day 0 for the Yakult-like Lc drink. The Yakult-like Lr drink had the minimum mean calcium content at T_5_ (8.28 ± 0.41 mg/100 g). The lowest sodium contents were observed on day 28 for the Yakult-like Lc drink (4.75 ± 0.22 mg/100 g) and Yakult-like Lr drink (4.35 ± 0.22 mg/100 g). At day 0, the highest values were recorded for both Yakult-like fermented milks at T_1_: 6.67 ± 0.33 mg/100 g for the Yakult-like Lc drink and 6.75 ± 0.33 mg/100g for the Yakult-like Lr drink. The maximum mean iron content was observed at T_5_ (14.11 ± 0.71 mg/100 g), followed by T_4_, T_3_, T_2_, and T_1_. At T_1_, the Yakult-like Lc drink had the lowest iron level (10.21 ± 0.51 mg/100 g) and a similar result was observed for the Yakult-like Lr drink. An increasing trend was observed: the minimum mean iron was observed at T_5_ (10.59 ± 0.09 mg/100 g) on day 0. At T_1_, the iron content was found to be highest at 10.69 ± 0.53 mg/100 g. The Yakult-like milk’s decreased mineral content may have been the result of use by microorganisms. The acidity of the Yakult-like fermented skimmed milks increased as the pH decreased. Since the minerals are unstable in an acidic environment, the mineral content in the fermented skimmed milk samples decreased as the acid concentration increased. Probiotic beverages made from different mixtures of milk analogs derived from African yam bean, soybean, and coconut produced comparable results. Except for the iron content, which increased, the contents of other minerals were found to decrease with fermentation and probiotic concentration [43]. A study of the mineral content (ppm) upon five treatments (control, T_1_, T_2_, T_3_, and T_4_) showed that as the added ratio of quinoa seed water extract (QSWE) increased, the mineral content of fermented quinoa beverages decreased. Calcium and phosphorus had the highest levels in both the control and other treatments, followed by zinc, magnesium, potassium, and sodium. With increasing added ratio of QSWE, the iron content increased in all treatments [44]. Mineral components are essential in milk products because an excess or deficiency can be harmful to human health. In a previous study of milk products (milk and yogurt), levels of the minerals calcium, magnesium, sodium, phosphorus, and iron were measured. In order of abundance, following calcium and potassium were phosphorus, sodium, magnesium, and iron [45].

### 3.5. Sensory Evaluation of Fermented Skimmed Milk Drinks

**Figure 1:**
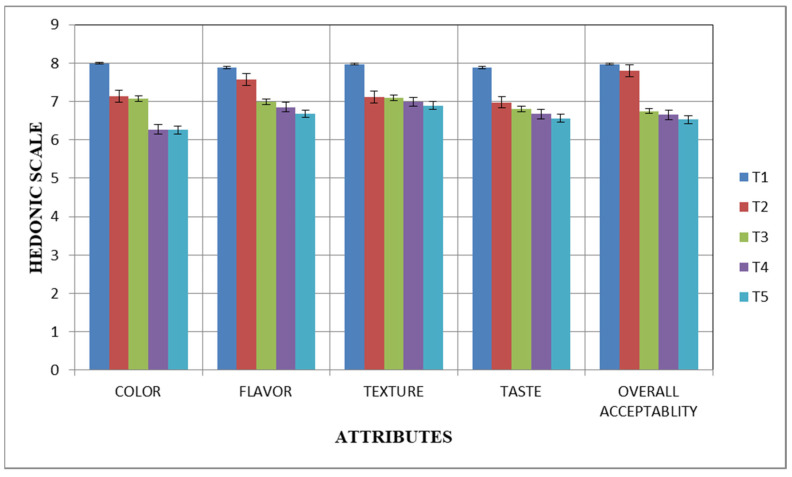
Sensory evaluation (mean value ± SD) of fermented skimmed milk drinks containing *Lacticaseibacillus casei* (Lc) at five different concentrations [1% (T_1_), 1.5% (T_2_), 2% (T_3_), 2.5% (T_4_), and 3% (T_5_)].

**Figure 2:**
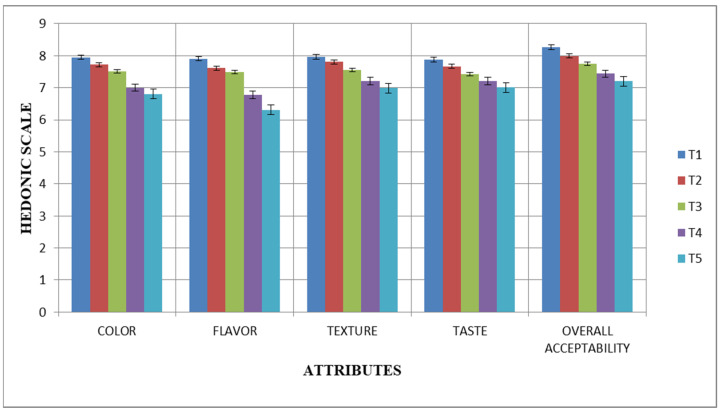
Sensory evaluation (mean value ± SD) of fermented skimmed milk drinks containing *Lacticaseibacillus rhamnosus* (Lr) at five different concentrations [1% (T_1_), 1.5% (T_2_), 2% (T_3_), 2.5% (T_4_), and 3% (T_5_)].

The graphs below illustrate how the fermented skimmed milk samples under study changed in terms of their sensory evaluation during the 28 days of cold storage at 4 °C (Figure 1 and Figure 2). The Yakult-like Lc drink and Yakult-like Lr drink were evaluated for flavor, taste, texture, color, and overall acceptability at five concentrations of cultures (T_1_, T_2_, T_3_, T_4_, and T_5_). The results showed that, among all of the treatments, T_1_ had the highest score on day 0 for flavor, taste, texture, color, and overall acceptability, followed by T_2_, T_3_, T_4_, and T_5_. On day 28 of storage, it was observed that the Yakult-like fermented skimmed milk samples at T_1_ (1% culture concentration) had the highest score. However, the sensory parameters of the Yakult-like Lc drink and Yakult-like Lr drink, in terms of flavor, taste, texture, color and overall acceptability, varied significantly (*p* ≤ 0.05). The analytical findings showed that all sensory parameters were significantly impacted by the different treatments that were used. The graphs demonstrate that all sensory parameters were significantly (*p* ≤ 0.05) impacted by the addition of the lactic acid bacteria (LAB) cultures.

The study conducted by Pato et al. [26] investigating the sensory analysis of fermented milk using probiotics showed that the addition of sucrose was found to have a significant effect (*p* ≤ 0.05). The use of 15% skimmed milk and variations in sucrose content had a significant impact on all sensory aspects, including color, taste, and texture. During cold storage, all sensory scores of the samples decreased. This might have been associated with the acidity and flavor of the bio-yogurt and acidophilus milk. Samples had an intense flavor and low acidity at the beginning of storage. However, they had higher acidity after 21 days, which resulted in lower organoleptic scores [35]. According to the sensory characteristics of fermented milks prepared with probiotic cultures and dairy starter cultures, no appreciable difference was observed between probiotics and dairy starters in terms of flavor, appearance, texture, and overall acceptability. Overall, it has been found that probiotic-enriched fermented milk has sensory qualities that are comparable to those prepared with commercial dairy starters [46]. Another study reported that there were no discernible differences in the sensory qualities of taste, texture, and overall acceptability. However, the sample’s aroma and appearance had observable differences [47].

## 4. Conclusions

In this study, Lc had a non-significant (*p* ≤ 0.05) effect on the moisture content of Yakult, while Lr had a significant (*p* ≤ 0.05) effect on the moisture content. Lc had a significant (*p* ≤ 0.05) effect on the total titratable acidity (TTA), while Lr had a non-significant (*p* ≤ 0.05) effect on the TTA of Yakult. Lc and Lr had significant effects on the protein content, carbohydrate content, total soluble solids (TSS), and viscosity, and non-significant effects on the fat content, ash content, and pH with increasing concentration of LAB in Yakult. In comparison, the Lr samples had slightly higher moisture contents, fat contents, and lacticaseibacillus viable counts than the Lc samples at all concentrations during the storage interval of 28 days, while no effect was observed in terms of the protein, ash, and carbohydrate contents. The antioxidant activities estimated by the DPPH, FRAP, and ABTS assays decreased over time, while those estimated by the TFC and TPC assays increased, and sensory evaluation revealed higher acceptability. Probiotics can be used in milk fermentation to create fermented dairy drinks and beverages. Yakult can be used to market an innovative beverage to a specific consumer group, producers, and industry, and scale retail distribution.

## Figures and Tables

**Table 1:** Experimental design for skimmed milk fermented with different concentrations of *Lacticaseibacillus casei* (Lc) and *Lacticaseibacillus rhamnosus* (Lr).

Treatment (T)	Levels of Lc and Lr (%)
T_1_	1
T_2_	1.5
T_3_	2
T_4_	2.5
T_5_	3

**Table 2:** Proximate composition (mean value ± SD) of fermented skimmed milk drinks containing *Lacticaseibacillus casei* (Lc) at five different concentrations [1% (T_1_), 1.5% (T_2_), 2% (T_3_), 2.5% (T_4_), and 3% (T_5_)] throughout 28 days of storage.

Time	Treatment	Moisture (%)	Fat (%)	Protein (%)	Ash (%)	Carbohydrate (%)
Day 0	T_1_	85.22 ± 0.14 ^a^	0.33 ± 0.21 ^a^	2.65 ± 0.28 ^c^	0.42 ± 0.11 ^a^	11.10 ± 0.11 ^a^
	T_2_	85.14 ± 0.13 ^a^	0.23 ± 0.22 ^a^	2.22 ± 0.28 ^c^	0.41 ± 0.11 ^a^	11.01 ± 0.11 ^a^
	T_3_	85.51 ± 0.13 ^a^	0.23 ± 0.22 ^a^	2.75 ± 0.28 ^c^	0.43 ± 0.11 ^a^	10.25 ± 0.11 ^b^
	T_4_	85.65 ± 0.13 ^a^	0.33 ± 0.21 ^a^	2.99 ± 0.29 ^c^	0.41 ± 0.11 ^a^	10.35 ± 0.11 ^b^
	T_5_	85.36 ± 0.13 ^a^	0.22 ± 0.22 ^a^	2.62 ± 0.28 ^c^	0.41 ± 0.11 ^a^	9.75 ± 0.11 ^c^
Day 7	T_1_	85.14 ± 0.13 ^a^	0.35 ± 0.20 ^a^	3.21 ± 0.28 ^b^	0.44 ± 0.11 ^a^	10.47 ± 0.11 ^a^
	T_2_	84.94 ± 0.13 ^b^	0.25 ± 0.22 ^a^	3.08 ± 0.28 ^b^	0.44 ± 0.11 ^a^	10.42 ± 0.11 ^a^
	T_3_	84.81 ± 0.10 ^b^	0.24 ± 0.22 ^a^	3.22 ± 0.28 ^b^	0.39 ± 0.10 ^a^	10.12 ± 0.10 ^a^
	T_4_	84.90 ± 0.10 ^b^	0.36 ± 0.21 ^a^	3.27 ± 0.29 ^b^	0.41 ± 0.11 ^a^	10.24 ± 0.11 ^a^
	T_5_	84.94 ± 0.12 ^b^	0.28 ± 0.21 ^a^	3.22 ± 0.28 ^b^	0.42 ± 0.10 ^a^	9.48 ± 0.10 ^b^
Day 14	T_1_	85.22 ± 0.13 ^a^	0.34 ± 0.19 ^a^	3.51 ± 0.27 ^b^	0.46 ± 0.10 ^a^	9.47 ± 0.10 ^b^
	T_2_	84.64 ± 0.11 ^b^	0.24 ± 0.21 ^a^	3.38 ± 0.28 ^b^	0.35 ± 0.10 ^a^	9.46 ± 0.10 ^b^
	T_3_	84.61 ± 0.09 ^b^	0.23 ± 0.21 ^a^	3.22 ± 0.28 ^b^	0.47 ± 0.10 ^a^	9.41 ± 0.10 ^b^
	T_4_	84.25 ± 0.09 ^b^	0.33 ± 0.20 ^a^	3.27 ± 0.29 ^b^	0.51 ± 0.10 ^a^	9.47 ± 0.10 ^b^
	T_5_	84.51 ± 0.12 ^b^	0.33 ± 0.20 ^a^	3.25 ± 0.27 ^b^	0.59 ± 0.10 ^a^	9.46 ± 0.10 ^b^
Day 21	T_1_	85.31 ± 0.10 ^a^	0.31 ± 0.19 ^a^	3.89 ± 0.25 ^b^	0.48 ± 0.10 ^a^	9.01 ± 0.10 ^b^
	T_2_	84.77 ± 0.09 ^b^	0.31 ± 0.21 ^a^	3.99 ± 0.27 ^b^	0.51 ± 0.10 ^a^	8.83 ± 0.10 ^c^
	T_3_	85.21 ± 0.08 ^a^	0.25 ± 0.20 ^a^	3.06 ± 0.29 ^b^	0.51 ± 0.09 ^a^	8.80 ± 0.09 ^c^
	T_4_	85.09 ± 0.08 ^a^	0.32 ± 0.20 ^a^	3.09 ± 0.30 ^b^	0.51 ± 0.09 ^a^	8.42 ± 0.09 ^c^
	T_5_	84.81 ± 0.09 ^b^	0.34 ± 0.20 ^a^	3.95 ± 0.26 ^b^	0.41 ± 0.09 ^a^	8.16 ± 0.09 ^c^
Day 28	T_1_	84.77 ± 0.09 ^b^	0.16 ± 0.18 ^a^	4.19 ± 0.24 ^a^	0.51 ± 0.09 ^a^	8.44 ± 0.09 ^c^
	T_2_	84.98 ± 0.08 ^b^	0.28 ± 0.20 ^a^	4.08 ± 0.25 ^a^	0.51 ± 0.10 ^a^	8.16 ± 0.10 ^c^
	T_3_	85.28 ± 0.08 ^a^	0.16 ± 0.19 ^a^	4.16 ± 0.27 ^a^	0.55 ± 0.09 ^a^	8.64 ± 0.09 ^c^
	T_4_	84.67 ± 0.08 ^b^	0.33 ± 0.19 ^a^	4.24 ± 0.27 ^a^	0.42 ± 0.09 ^a^	7.56 ± 0.09 ^d^
	T_5_	85.41 ± 0.08 ^a^	0.34 ± 0.19 ^a^	4.18 ± 0.25 ^a^	0.48 ± 0.09 ^a^	7.43 ± 0.09 ^d^

Note: Values with different letters in the same column are significantly different at *p* ≤ 0.05. SD, standard deviation.

**Table 3:** Proximate composition (mean value ± SD) of fermented skimmed milk drinks containing *Lacticaseibacillus rhamnosus* (Lr) at five different concentrations [1% (T_1_), 1.5% (T_2_), 2% (T_3_), 2.5% (T_4_), and 3% (T_5_)] throughout 28 days of storage.

Time	Treatment	Moisture (%)	Fat (%)	Protein (%)	Ash (%)	Carbohydrate (%)
Day 0	T_1_	88.65 ± 0.14 ^a^	0.29 ± 0.21 ^a^	2.96 ± 0.28 ^c^	0.49 ± 0.11 ^a^	11.28 ± 0.11 ^a^
	T_2_	88.45 ± 0.13 ^a^	0.28 ± 0.22 ^a^	2.91 ± 0.28 ^c^	0.44 ± 0.11 ^a^	11.85 ± 0.11 ^a^
	T_3_	88.67 ± 0.13 ^a^	0.25 ± 0.22 ^a^	2.85 ± 0.28 ^c^	0.48 ± 0.11 ^a^	10.63 ± 0.11 ^b^
	T_4_	87.65 ± 0.13 ^b^	0.28 ± 0.21 ^a^	2.82 ± 0.29 ^c^	0.42 ± 0.11 ^a^	10.15 ± 0.11 ^b^
	T_5_	87.85 ± 0.13 ^b^	0.29 ± 0.21 ^a^	2.92 ± 0.28 ^c^	0.41 ± 0.11 ^a^	9.75 ± 0.11 ^c^
Day 7	T_1_	88.45 ± 0.13 ^a^	0.26 ± 0.20 ^a^	2.85 ± 0.28 ^c^	0.45 ± 0.11 ^a^	10.25 ± 0.11 ^b^
	T_2_	88.45 ± 0.13 ^a^	0.24 ± 0.22 ^a^	2.82 ± 0.28 ^c^	0.46 ± 0.11 ^a^	10.75 ± 0.11 ^b^
	T_3_	88.62 ± 0.10 ^a^	0.23 ± 0.22 ^a^	2.81 ± 0.28 ^c^	0.49 ± 0.10 ^a^	10.12 ± 0.10 ^b^
	T_4_	87.55 ± 0.10 ^b^	0.27 ± 0.21 ^a^	2.79 ± 0.29 ^c^	0.44 ± 0.11 ^a^	9.46 ± 0.11 ^c^
	T_5_	86.70 ± 0.10 ^c^	0.23 ± 0.21 ^a^	3.21 ± 0.28 ^b^	0.43 ± 0.10 ^a^	9.51 ± 0.10 ^c^
Day 14	T_1_	88.75 ± 0.13 ^a^	0.25 ± 0.19 ^a^	3.21 ± 0.27 ^b^	0.51 ± 0.10 ^a^	9.51 ± 0.10 ^c^
	T_2_	87.75 ± 0.11 ^b^	0.24 ± 0.21 ^a^	3.21 ± 0.28 ^b^	0.47 ± 0.10 ^a^	9.47 ± 0.10 ^c^
	T_3_	87.41 ± 0.09 ^b^	0.32 ± 0.21 ^a^	3.22 ± 0.28 ^b^	0.51 ± 0.10 ^a^	9.45 ± 0.10 ^c^
	T_4_	87.40 ± 0.09 ^b^	0.45 ± 0.20 ^a^	3.21 ± 0.29 ^b^	0.47 ± 0.10 ^a^	8.83 ± 0.10 ^d^
	T_5_	85.41 ± 0.09 ^d^	0.45 ± 0.20 ^a^	3.51 ± 0.30 ^b^	0.46 ± 0.10 ^a^	8.82 ± 0.10 ^d^
Day 21	T_1_	88.15 ± 0.10 ^a^	0.24 ± 0.19 ^a^	3.31 ± 0.25 ^b^	0.51 ± 0.10 ^a^	9.01 ± 0.10 ^c^
	T_2_	87.42 ± 0.09 ^b^	0.23 ± 0.21 ^a^	3.15 ± 0.27 ^b^	0.51 ± 0.10 ^a^	9.41 ± 0.10 ^c^
	T_3_	87.40 ± 0.08 ^b^	0.31 ± 0.20a	3.21 ± 0.29 ^b^	0.88 ± 0.09 ^a^	8.42 ± 0.09 ^d^
	T_4_	86.35 ± 0.08 ^c^	0.24 ± 0.20 ^a^	3.51 ± 0.30 ^b^	0.52 ± 0.09 ^a^	8.16 ± 0.09 ^d^
	T_5_	84.74 ± 0.08^e^	0.23 ± 0.20 ^a^	3.79 ± 0.26 ^b^	0.51 ± 0.09 ^a^	8.52 ± 0.09 ^d^
Day 28	T_1_	87.70 ± 0.09 ^b^	0.21 ± 0.18 ^a^	4.09 ± 0.24 ^a^	0.53 ± 0.09 ^a^	8.42 ± 0.10 ^d^
	T_2_	87.69 ± 0.08 ^b^	0.27 ± 0.20 ^a^	4.08 ± 0.25 ^a^	0.51 ± 0.10 ^a^	8.16 ± 0.10 ^d^
	T_3_	87.82 ± 0.08 ^b^	0.31 ± 0.19 ^a^	4.11 ± 0.27 ^a^	0.53 ± 0.09 ^a^	8.21 ± 0.09 ^d^
	T_4_	86.31 ± 0.08 ^c^	0.31 ± 0.19 ^a^	4.12 ± 0.27 ^a^	0.51 ± 0.09 ^a^	7.44 ± 0.09 ^e^
	T_5_	84.24 ± 0.08 ^e^	0.31 ± 0.19 ^a^	4.09 ± 0.27 ^a^	0.52 ± 0.09 ^a^	7.43 ± 0.09 ^e^

Note: Values with different letters in the same column are significantly different at *p* ≤ 0.05. SD, standard deviation.

**Table 4:** Physiochemical composition (mean value ± SD) of fermented skimmed milk drinks containing Lacticaseibacillus casei (Lc) at five different concentrations [1% (T_1_), 1.5% (T_2_), 2% (T_3_), 2.5% (T_4_), and 3% (T_5_)] throughout 28 days of storage.

Time	Treatment	TSS (%)	TTA (%)	pH (%)	Viscosity (%)
Day 0	T_1_	10.58 ± 0.52 ^e^	0.27 ± 0.01 ^b^	4.49 ± 0.22 ^a^	10.64 ± 0.11 ^f^
	T_2_	10.81 ± 0.54 ^e^	0.27 ± 0.01 ^b^	4.38 ± 0.22 ^a^	10.74 ± 0.11 ^f^
	T_3_	10.63 ± 0.53 ^e^	0.28 ± 0.01 ^b^	4.38 ± 0.21 ^a^	10.82 ± 0.11 ^f^
	T_4_	10.45 ± 0.52 ^e^	0.27 ± 0.01 ^b^	4.44 ± 0.21 ^a^	11.95 ± 0.11 ^e^
	T_5_	11.18 ± 0.55 ^d^	0.27 ± 0.01 ^b^	4.33 ± 0.22 ^a^	12.43 ± 0.11 ^d^
Day 7	T_1_	11.35 ± 0.56 ^d^	0.28 ± 0.01 ^b^	4.31 ± 0.21 ^a^	13.64 ± 0.11 ^c^
	T_2_	11.18 ± 0.55 ^d^	0.28 ± 0.01 ^b^	4.59 ± 0.22 ^a^	13.51 ± 0.11 ^c^
	T_3_	11.33 ± 0.55 ^d^	0.20 ± 0.01 ^b^	4.46 ± 0.21 ^a^	14.31 ± 0.10 ^b^
	T_4_	11.17 ± 0.54 ^d^	0.29 ± 0.01 ^b^	4.54 ± 0.21 ^a^	14.25 ± 0.11 ^b^
	T_5_	11.12 ± 0.54 ^d^	0.29 ± 0.01 ^b^	4.46 ± 0.22 ^b^	14.73 ± 0.10 ^b^
Day 14	T_1_	12.72 ± 0.63 ^c^	0.26 ± 0.01 ^b^	4.46 ± 0.22 ^a^	13.59 ± 0.10 ^c^
	T_2_	13.41 ± 0.66 ^b^	0.24 ± 0.01 ^b^	4.43 ± 0.21 ^a^	13.39 ± 0.10 ^c^
	T_3_	13.53 ± 0.65 ^b^	1.27 ± 0.06 ^a^	4.42 ± 0.21 ^a^	14.31 ± 0.10 ^b^
	T_4_	13.48 ± 0.65 ^b^	1.16 ± 0.05 ^a^	4.41 ± 0.21 ^a^	15.79 ± 0.10 ^a^
	T_5_	13.58 ± 0.64 ^b^	1.15 ± 0.05 ^a^	4.42 ± 0.21 ^a^	15.12 ± 0.10 ^a^
Day 21	T_1_	12.83 ± 0.64 ^c^	1.14 ± 0.05 ^a^	4.38 ± 0.21 ^a^	13.93 ± 0.10 ^c^
	T_2_	13.83 ± 0.63 ^b^	1.16 ± 0.05 ^a^	4.36 ± 0.21 ^a^	13.16 ± 0.10 ^c^
	T_3_	13.88 ± 0.65 ^b^	1.11 ± 0.05 ^a^	4.35 ± 0.21 ^a^	14.41 ± 0.09 ^b^
	T_4_	13.81 ± 0.64 ^b^	1.23 ± 0.05 ^a^	4.34 ± 0.21 ^a^	15.65 ± 0.09 ^a^
	T_5_	13.81 ± 0.64 ^b^	1.37 ± 0.05 ^a^	3.35 ± 0.16 ^b^	15.17 ± 0.09 ^a^
Day 28	T_1_	13.87 ± 0.69 ^b^	1.32 ± 0.05 ^a^	3.34 ± 0.16 ^b^	14.34 ± 0.09 ^b^
	T_2_	13.85 ± 0.68 ^b^	1.34 ± 0.15 ^a^	3.33 ± 0.17 ^b^	14.88 ± 0.10 ^b^
	T_3_	13.84 ± 0.68 ^b^	1.22 ± 0.05 ^a^	3.27 ± 0.17 ^b^	14.77 ± 0.09 ^b^
	T_4_	14.22 ± 0.68 ^a^	1.11 ± 0.05 ^a^	3.19 ± 0.17 ^b^	15.22 ± 0.09 ^a^
	T_5_	14.42 ± 0.69 ^a^	1.04 ± 0.04 ^a^	3.05 ± 0.15 ^b^	15.41 ± 0.09 ^a^

Note: Values with different letters in the same column are significantly different at *p* ≤ 0.05. SD, standard deviation; TSS, total soluble solids; TTA, total titratable acidity.

**Table 5:** Physicochemical composition (mean value ± SD) of fermented skimmed milk drinks containing *Lacticaseibacillus rhamnosus* (Lr) at five different concentrations [1% (T_1_), 1.5% (T_2_), 2% (T_3_), 2.5% (T_4_), and 3% (T_5_)] throughout 28 days of storage.

Time	Treatment	TSS (%)	TTA (%)	pH (%)	Viscosity (%)
Day 0	T_1_	11.35 ± 0.56 ^a^	0.27 ± 0.01 ^a^	4.49 ± 0.22 ^a^	11.41 ± 0.02 ^e^
	T_2_	11.08 ± 0.55 ^a^	0.27 ± 0.01 ^a^	4.48 ± 0.22 ^a^	11.44 ± 0.02 ^e^
	T_3_	10.92 ± 0.54 ^b^	0.28 ± 0.01 ^a^	4.45 ± 0.22 ^a^	11.130 ± 0.01 ^e^
	T_4_	11.26 ± 0.55 ^a^	0.24 ± 0.01 ^a^	3.84 ± 0.17 ^b^	12.24 ± 0.01 ^d^
	T_5_	10.21 ± 0.55 ^b^	0.21 ± 0.01 ^a^	3.71 ± 0.11 ^b^	12.23 ± 0.01 ^d^
Day 7	T_1_	11.31 ± 0.55 ^a^	0.22 ± 0.01 ^a^	4.48 ± 0.22 ^a^	12.46 ± 0.02 ^d^
	T_2_	11.02 ± 0.55 ^a^	0.26 ± 0.01 ^a^	4.46 ± 0.22 ^a^	12.45 ± 0.02 ^d^
	T_3_	10.89 ± 0.54 ^b^	0.27 ± 0.01 ^a^	3.44 ± 0.26 ^b^	12.45 ± 0.10 ^d^
	T_4_	10.62 ± 0.54 ^b^	0.28 ± 0.01 ^a^	3.33 ± 0.16 ^b^	13.34 ± 0.01 ^c^
	T_5_	10.71 ± 0.55 ^b^	0.29 ± 0.01 ^a^	3.31 ± 0.16 ^b^	13.31 ± 0.01 ^c^
Day 14	T_1_	11.26 ± 0.56 ^a^	0.26 ± 0.01 ^a^	4.46 ± 0.22 ^a^	13.44 ± 0.02 ^c^
	T_2_	10.96 ± 0.56 ^b^	0.21 ± 0.01 ^a^	4.43 ± 0.21 ^a^	13.41 ± 0.02 ^c^
	T_3_	10.78 ± 0.55 ^b^	0.11 ± 0.009 ^a^	4.42 ± 0.21 ^a^	13.35 ± 0.01 ^c^
	T_4_	10.62 ± 0.55 ^b^	0.11 ± 0.009 ^a^	4.41 ± 0.20 ^a^	14.33 ± 0.01 ^b^
	T_5_	10.48 ± 0.54 ^b^	0.11 ± 0.009 ^a^	3.91 ± 0.17 ^b^	14.21 ± 0.01 ^b^
Day 21	T_1_	11.22 ± 0.56 ^a^	0.29 ± 0.01 ^a^	4.41 ± 0.10 ^a^	14.43 ± 0.02 ^b^
	T_2_	10.96 ± 0.53 ^b^	0.27 ± 0.01 ^a^	4.36 ± 0.21 ^a^	14.44 ± 0.02 ^b^
	T_3_	10.78 ± 0.51 ^b^	0.26 ± 0.01 ^a^	4.35 ± 0.21 ^a^	14.39 ± 0.01 ^b^
	T_4_	10.62 ± 0.54 ^b^	0.19 ± 0.008 ^a^	4.34 ± 0.21 ^a^	15.31 ± 0.01 ^a^
	T_5_	10.48 ± 0.53 ^b^	0.13 ± 0.006 ^a^	3.97 ± 0.18 ^b^	15.24 ± 0.01 ^a^
Day 28	T_1_	11.41 ± 0.55 ^a^	0.29 ± 0.01 ^a^	4.35 ± 0.21 ^a^	14.41 ± 0.02 ^b^
	T_2_	10.71 ± 0.54 ^b^	0.27 ± 0.01 ^a^	4.23 ± 0.10 ^a^	14.40 ± 0.02 ^b^
	T_3_	10.57 ± 0.54 ^b^	0.14 ± 0.01 ^a^	4.31 ± 0.21 ^a^	15.38 ± 0.01 ^a^
	T_4_	10.52 ± 0.54 ^b^	0.12 ± 0.01 ^a^	4.28 ± 0.20 ^a^	15.39 ± 0.01 ^a^
	T_5_	10.45 ± 0.55 ^b^	0.11 ± 0.006 ^a^	3.76 ± 0.18 ^b^	15.28 ± 0.01 ^a^

Note: Values with different letters in the same column are significantly different at *p* ≤ 0.05. SD, standard deviation; TSS, total soluble solids; TTA, total titratable acidity.

**Table 6:** Microbial viable counts [log (CFU/mL)] (mean value ± SD) of *Lacticaseibacillus casei* (Lc) and *Lacticaseibacillus rhamnosus* (Lr) in fermented skimmed milk drinks at five different concentrations [1% (T_1_), 1.5% (T_2_), 2% (T_3_), 2.5% (T_4_), and 3% (T_5_)] throughout 28 days of storage.

Time	Treatment	Lc (Log CFU/mL)	Lr (Log CFU/mL)
Day 0	T_1_	5.74 ± 0.28 ^b^	7.34 ± 0.36 ^b^
	T_2_	5.67 ± 0. 28 ^b^	7.31 ± 0.36 ^b^
	T_3_	5.61 ± 0.28 ^b^	7.28 ± 0.36l ^b^
	T_4_	6.06 ± 0.30 ^a^	7.21 ± 0.36 ^b^
	T_5_	6.14 ± 0.30 ^e^	7.16 ± 0.35 ^b^
Day 7	T_1_	6.51 ± 0.32 ^a^	7.63 ± 0.38 ^b^
	T_2_	6.62 ± 0.32 ^a^	7.60 ± 0.38 ^b^
	T_3_	6.64 ± 0.33 ^a^	7.59 ± 0.37 ^b^
	T_4_	6.67 ± 0.33 ^a^	7.57 ± 0.37 ^b^
	T_5_	6.73 ± 0.33 ^a^	7.55 ± 0.27 ^b^
Day 14	T_1_	6.45 ± 0.32 ^a^	8.29 ± 0.41 ^a^
	T_2_	6.51 ± 0.30 ^a^	8.25 ± 0.41 ^a^
	T_3_	6.19 ± 0.30 ^a^	8.21 ± 0.41 ^a^
	T_4_	6.57 ± 0.32 ^a^	8.25 ± 0.41 ^a^
	T_5_	6.63 ± 0.33 ^a^	8.19 ± 0.41 ^a^
Day 21	T_1_	6.36 ± 0.31 ^a^	8.12 ± 0.40 ^a^
	T_2_	6.42 ± 0.32 ^a^	8.65 ± 0.43 ^a^
	T_3_	6.54 ± 0.32 ^a^	8.61 ± 0.43 ^a^
	T_4_	6.57 ± 0.32 ^a^	8.57 ± 0.43 ^a^
	T_5_	6.63 ± 0.33 ^a^	8.52 ± 0.42 ^a^
Day 28	T_1_	6.34 ± 0.31 ^a^	8.69 ± 0.43 ^a^
	T_2_	6.41 ± 0.32 ^a^	8.67 ± 0.43 ^a^
	T_3_	6.42 ± 0.32 ^a^	8.63 ± 0.43 ^a^
	T_4_	6.52 ± 0.32 ^a^	8.59 ± 0.42 ^a^
	T_5_	6.58 ± 0.32 ^a^	8.42 ± 0.42 ^a^

Note: Values with different letters in the same column are significantly different at *p* ≤ 0.05. SD, standard deviation.

**Table 7:** Antioxidant activity (mean value ± SD) of fermented skimmed milk drinks containing *Lacticaseibacillus casei* (Lc) at five different concentrations [1% (T_1_), 1.5% (T_2_), 2% (T_3_), 2.5% (T_4_), and 3% (T_5_)] throughout 28 days of storage.

Time	Treatment	DPPH (%)	FRAP (mmol Fe^2+^/L)	ABTS (%)	TPC (mg_GAE_/mL)	TFC (mg_QE_/mL)
Day 0	T_1_	88.65 ± 0.14 ^a^	0.23 ± 0.01 ^a^	52.38 ± 2.61^o^	4.61 ± 0.23 ^a^	0.21 ± 0.01 ^a^
	T_2_	88.45 ± 0.13 ^a^	0.45 ± 0.02 ^a^	74.23 ± 3.71 ^g^	4.63 ± 0.23 ^a^	0.22 ± 0.01 ^a^
	T_3_	88.67 ± 0.13 ^a^	0.64 ± 0.03 ^a^	78.24 ± 3.72 ^d^	4.29 ± 0.21 ^a^	0.12 ± 0.01 ^a^
	T_4_	87.65 ± 0.13 ^b^	0.68 ± 0.03 ^a^	83.17 ± 4.15 ^b^	4.21 ± 0.21 ^a^	0.14 ± 0.01 ^a^
	T_5_	87.85 ± 0.13 ^b^	0.71 ± 0.03 ^a^	84.69 ± 4.15 ^a^	4.08 ± 0.20 ^a^	0.10 ± 0.01 ^a^
Day 7	T_1_	88.45 ± 0.13 ^a^	0.31 ± 0.01 ^a^	50.63 ± 2.53^p^	4.62 ± 0.23 ^a^	0.22 ± 0.01 ^a^
	T_2_	88.45 ± 0.13 ^a^	0.55 ± 0.02 ^a^	54.93 ± 2.74^n^	4.52 ± 0.22 ^a^	0.23 ± 0.01 ^a^
	T_3_	88.62 ± 0.10 ^a^	0.61 ± 0.03 ^a^	71.38 ± 3.56^k^	4.44 ± 0.22 ^a^	0.24 ± 0.01 ^a^
	T_4_	87.55 ± 0.10 ^b^	0.62 ± 0.03 ^a^	75.15 ± 3.75 ^f^	4.31 ± 0.21 ^a^	0.22 ± 0.01 ^a^
	T_5_	86.70 ± 0.10 ^c^	0.64 ± 0.03 ^a^	80.12 ± 4.01 ^c^	4.19 ± 0.20 ^a^	0.14 ± 0.01 ^a^
Day 14	T_1_	88.75 ± 0.13 ^a^	0.23 ± 0.01 ^a^	45.36 ± 2.26 ^r^	4.65 ± 0.23 ^a^	0.33 ± 0.01 ^a^
	T_2_	87.75 ± 0.11 ^b^	0.54 ± 0.02 ^a^	52.13 ± 2.70 ^o^	4.63 ± 0.23 ^a^	0.24 ± 0.01 ^a^
	T_3_	87.41 ± 0.09 ^b^	0.61 ± 0.03 ^a^	69.25 ± 3.46 ^l^	4.42 ± 0.22 ^a^	0.27 ± 0.01 ^a^
	T_4_	87.40 ± 0.09 ^b^	0.64 ± 0.03 ^a^	71.26 ± 3.56 ^k^	4.32 ± 0.21 ^a^	0.24 ± 0.01 ^a^
	T_5_	85.41 ± 0.09 ^d^	0.69 ± 0.03 ^a^	71.57 ± 3.56 ^k^	4.22 ± 0.21 ^a^	0.28 ± 0.01 ^a^
Day 21	T_1_	88.15 ± 0.10 ^a^	0.18 ± 0.09 ^a^	39.31 ± 1.96 ^t^	4.62 ± 0.23 ^a^	0.27 ± 0.01 ^a^
	T_2_	87.42 ± 0.09 ^b^	0.52 ± 0.02 ^a^	47.26 ± 2.36 ^q^	4.63 ± 0.23 ^a^	0.35 ± 0.01 ^a^
	T_3_	87.40 ± 0.08 ^b^	0.59 ± 0.02 ^a^	62.16 ± 3.10 ^l^	4.45 ± 0.22 ^a^	0.31 ± 0.01 ^a^
	T_4_	86.35 ± 0.08 ^c^	0.64 ± 0.03 ^a^	67.37 ± 3.36 ^n^	4.31 ± 0.21 ^a^	0.31 ± 0.01 ^a^
	T_5_	84.74 ± 0.08 ^e^	0.67 ± 0.03 ^a^	68.37 ± 3.46 ^m^	4.25 ± 0.21 ^a^	0.32 ± 0.01 ^a^
Day 28	T_1_	87.70 ± 0.09 ^b^	0.13 ± 0.06 ^a^	31.43 ± 1.57 ^u^	4.66 ± 0.23 ^a^	0.28 ± 0.01 ^a^
	T_2_	87.69 ± 0.08 ^b^	0.53 ± 0.02 ^a^	42.26 ± 02.13 ^s^	4.64 ± 0.23 ^a^	0.33 ± 0.01 ^a^
	T_3_	87.82 ± 0.08 ^b^	0.61 ± 0.03 ^a^	59.81 ± 2.13 ^m^	4.47 ± 0.22 ^a^	0.31 ± 0.01 ^a^
	T_4_	86.31 ± 0.08 ^c^	0.65 ± 0.03 ^a^	69.17 ± 3.12 ^l^	4.28 ± 0.21 ^a^	0.35 ± 0.01 ^a^
	T_5_	84.24 ± 0.08 ^e^	0.69 ± 0.03 ^a^	72.14 ± 3.12 ^h^	4.15 ± 0.21 ^a^	0.37 ± 0.01 ^a^

Note: Values with different letters in the same column are significantly different at *p* ≤ 0.05. SD, standard deviation; DPPH, 2, 2-diphenyl-1-picrylhydrazyl; FRAP, ferric-reducing antioxidant power; ABTS, 2, 29-Azinobis (3-ethylene benzothiazoline) 6-sulphonic acid; TPC, total phenolic content; TFC, total flavonoid content.

**Table 8:** Antioxidant activity (mean value ± SD) of fermented skimmed milk drinks containing *Lacticaseibacillus rhamnosus* (Lr) at five different concentrations [1% (T_1_), 1.5% (T_2_), 2% (T_3_), 2.5% (T_4_), and 3% (T_5_)] throughout 28 days of storage.

Time	Treatment	DPPH (%)	FRAP (mmol Fe^2+^/L)	ABTS (%)	TPC (mg _GAE_/mL)	TFC (mg _QE_/mL)
Day 0	T_1_	30.61 ± 1.53^h^	0.29 ± 1.48 ^f^	58.23 ± 2.61^q^	3.38 ± 0.16 ^c^	1.22 ± 0.06 ^c^
	T_2_	35.86 ± 1.79 ^f^	0.22 ± 1.11 ^f^	76.72 ± 3.86^h^	2.79 ± 0.13 ^c^	1.54 ± 0.17 ^c^
	T_3_	39.61 ± 1.98 ^f^	1.26 ± 1.33 ^e^	79.72 ± 3.71 ^e^	3.79 ± 0. 18 ^c^	2.47 ± 0.16 ^b^
	T_4_	35.24 ± 1.76 ^g^	1.275 ± 1.33 ^e^	84.46 ± 4.15 ^b^	4.16 ± 0.20 ^b^	2.58 ± 0.12 ^b^
	T_5_	40.22 ± 2.01 ^f^	1.25 ± 1.33 ^e^	87.69 ± 4.15 ^a^	4.89 ± 0.25 ^b^	3.18 ± 0.15 ^a^
Day 7	T_1_	42.15 ± 2.10 ^e^	1.36 ± 1.84 ^e^	54.83 ± 2.74^r^	3.18 ± 0.15 ^c^	1.85 ± 0.09 ^c^
	T_2_	52.81 ± 2.64 ^a^	1.35 ± 1.76 ^e^	73.86 ± 3.69 ^b^	3.25 ± 0.17 ^c^	2.33 ± 0.17 ^b^
	T_3_	53.58 ± 2.67 ^a^	2.35 ± 1.78 ^d^	15.91 ± 3.13^j^	4.22 ± 0.21 ^b^	2.54 ± 0.15 ^b^
	T_4_	57.76 ± 2.88 ^a^	2.34 ± 1.71 ^d^	82.31 ± 3.13 ^d^	4.42 ± 0. 21 ^b^	3.38 ± 0.16 ^a^
	T_5_	59.94 ± 2.99 ^a^	2.32 ± 1.62 ^d^	80.37 ± 4.13 ^d^	5.09 ± 0.26 ^a^	3.43 ± 0.17 ^a^
Day 14	T_1_	42.14 ± 2.10 ^e^	2.57 ± 2.88 ^d^	52.10 ± 2.60^s^	3.37 ± 0.16 ^c^	1.89 ± 0.09 ^c^
	T_2_	46.13 ± 2.30 ^b^	2.56 ± 2.88 ^c^	71.67 ± 3.58 ^b^	3.21 ± 0.17 ^c^	1.54 ± 0.16 ^c^
	T_3_	52.47 ± 2.62 ^a^	3.56 ± 2.88 ^b^	73.05 ± 3.15^k^	4.21 ± 0.21 ^b^	2.47 ± 0.15 ^b^
	T_4_	53.16 ± 2.65 ^a^	3.55 ± 2.75 ^b^	78.37 ± 3.13 ^f^	4.16 ± 0.20 ^b^	2.58 ± 0.12 ^b^
	T_5_	58.27 ± 2.91 ^a^	3.53 ± 2.67 ^b^	80.31 ± 4.13 ^d^	5.76 ± 0.28 ^a^	3.43 ± 0.17 ^a^
Day 21	T_1_	42.51 ± 2.10 ^a^	3.57 ± 2.88 ^b^	47.16 ± 2.35^u^	3.09 ± 0.15 ^c^	1.11 ± 0.05 ^c^
	T_2_	46.38 ± 2.30 ^c^	3.56 ± 2.88 ^b^	76.62 ± 3.83 ^d^	3.87 ± 0.19 ^c^	1.55 ± 0.16 ^c^
	T_3_	52.28 ± 2.62 ^a^	3.56 ± 2.85 ^b^	70.69 ± 3.53 ^n^	4.45 ± 0.21 ^b^	2.33 ± 0.14 ^b^
	T_4_	53.41 ± 2.65 ^a^	4.56 ± 2.85 ^a^	74.56 ± 3.43 ^i^	4.31 ± 0.21 ^b^	2.79 ± 0.12 ^b^
	T_5_	53.84 ± 2.69 ^a^	4.86 ± 2.80 ^a^	77.39 ± 3.63 ^e^	5.97 ± 0.29 ^a^	3.59 ± 0.17 ^a^
Day 28	T_1_	42.46 ± 2.10 ^d^	3.65 ± 3.28 ^b^	48.57 ± 2.42 ^t^	3.18 ± 0.15 ^c^	1.88 ± 0.09 ^c^
	T_2_	46.48 ± 2.30 ^b^	3.59 ± 2.95 ^b^	67.37 ± 3.36 ^e^	3.38 ± 0.19 ^c^	1.88 ± 0.15 ^c^
	T_3_	52.51 ± 2.62 ^a^	4.56 ± 2.84 ^a^	72.14 ± 3.60 ^l^	4.15 ± 0.20 ^b^	2.54 ± 0.13 ^b^
	T_4_	53.86 ± 2.65 ^a^	4.51 ± 2.57 ^a^	77.39 ± 3.86 ^e^	5.21 ± 0.26 ^a^	3.42 ± 0.14 ^a^
	T_5_	54.13 ± 2.70 ^a^	4.53 ± 2.67 ^a^	84.46 ± 4.22 ^b^	5.79 ± 0.26 ^a^	3.51 ± 0.17 ^a^

Note: Values with different letters in the same column are significantly different at *p* ≤ 0.05. SD, standard deviation; DPPH, 2, 2-diphenyl-1-picrylhydrazyl; FRAP, ferric-reducing antioxidant power; ABTS, 2, 29-Azinobis (3-ethylene benzothiazoline) 6-sulphonic acid; TPC, total phenolic content; TFC, total flavonoid content.

**Table 9:** Mineral content (mean value ± SD) of fermented skimmed milk drinks containing *Lacticaseibacillus casei* (Lc) at five different concentrations [1% (T_1_), 1.5% (T_2_), 2% (T_3_), 2.5% (T_4_), and 3% (T_5_)] throughout 28 days of storage.

Time	Treatment	Calcium (mg/100 g)	Sodium (mg/100 g)	Iron (mg/100 g)
Day 0	T_1_	9.64 ± 0.48 ^a^	6.67 ± 0.33 ^a^	10.21 ± 0.51 ^e^
	T_2_	9.61 ± 0.48 ^a^	6.65 ± 0.33 ^a^	10.24 ± 0.11 ^e^
	T_3_	9.55 ± 0.47 ^a^	6.63 ± 0.33 ^a^	10.21 ± 0.11 ^e^
	T_4_	9.51 ± 0.47 ^a^	6.61 ± 0.33 ^a^	10.18 ± 0.11 ^e^
	T_5_	9.48 ± 0.47 ^a^	6.23 ± 0.31 ^b^	11.17 ± 0.55 ^d^
Day 7	T_1_	8.85 ± 0.44 ^b^	6.87 ± 0.34 ^b^	11.15 ± 0.55 ^d^
	T_2_	8.82 ± 0.44 ^b^	6.85 ± 0.34 ^b^	11.12 ± 0.55 ^d^
	T_3_	8.53 ± 0.42 ^b^	6.82 ± 0.34 ^b^	11.11 ± 0.55 ^d^
	T_4_	8.79 ± 0.43 ^b^	6.78 ± 0.33 ^b^	11.09 ± 0.55 ^d^
	T_5_	8.75 ± 0.43 ^b^	6.74 ± 0.33 ^b^	12.36 ± 0.61 ^c^
Day 14	T_1_	7.47 ± 0.37 ^c^	5.97 ± 0.29 ^c^	12.34 ± 0.61 ^c^
	T_2_	7.45 ± 0.37 ^c^	5.95 ± 0.29 ^c^	12.32 ± 0.61 ^c^
	T_3_	7.43 ± 0.37 ^c^	5.92 ± 0.29 ^bc^	12.31 ± 0.61 ^c^
	T_4_	7.41 ± 0.37 ^c^	5.89 ± 0.29 ^bc^	12.28 ± 0.61 ^c^
	T_5_	7.31 ± 0.36 ^c^	5.86 ± 0.29 ^c^	13.39 ± 0.66 ^b^
Day 21	T_1_	7.25 ± 0.36 ^c^	5.84 ± 0.29 ^c^	13.37 ± 0.66 ^b^
	T_2_	7.23 ± 0.36 ^c^	5.82 ± 0.29 ^c^	13.35 ± 0.66 ^b^
	T_3_	7.21 ± 0.36 ^c^	5.79 ± 0.28 ^c^	13.32 ± 0.66 ^b^
	T_4_	7.18 ± 0.35 ^c^	5.75 ± 0.28 ^c^	13.31 ± 0.66 ^b^
	T_5_	6.64 ± 0.33 ^d^	5.72 ± 0.28 ^c^	14.14 ± 0.70 ^a^
Day 28	T_1_	6.36 ± 0.33 ^d^	4.87 ± 0.24 ^d^	14.21 ± 0.71 ^a^
	T_2_	6.33 ± 0.33 ^d^	4.85 ± 0.24 ^d^	14.18 ± 0.71 ^a^
	T_3_	6.31 ± 0.33 ^d^	4.82 ± 0.24 ^d^	14.15 ± 0.71 ^a^
	T_4_	6.28 ± 0.31 ^d^	4.78 ± 0.23 ^d^	14.12 ± 0.71 ^a^
	T_5_	6.26 ± 0.31 ^d^	4.75 ± 0.23 ^d^	14.11 ± 0.71 ^a^

Note: Values with different letters in the same column are significantly different at *p* ≤ 0.05. SD, standard deviation.

**Table 10:** Mineral content (mean value ± SD) of fermented skimmed milk drinks containing *Lacticaseibacillus rhamnosus* (Lr) at five different concentrations [1% (T_1_), 1.5% (T_2_), 2% (T_3_), 2.5% (T_4_), and 3% (T_5_)] throughout 28 days of storage.

Time	Treatment	Calcium (mg/100 g)	Sodium (mg/100 g)	Iron (mg/100 g)
Day 0	T_1_	8.37 ± 0.41 ^a^	6.74 ± 0.33 ^a^	10.69 ± 0.53 ^e^
	T_2_	8.35 ± 0.41 ^a^	6.75 ± 0.33 ^a^	10.66 ± 0.53 ^e^
	T_3_	8.33 ± 0.41 ^a^	6.73 ± 0.33 ^a^	10.64 ± 0.53 ^e^
	T_4_	8.32 ± 0.41 ^a^	6.71 ± 0.33 ^a^	10.61 ± 0.53 ^e^
	T_5_	8.28 ± 0.41 ^a^	6.68 ± 0.33 ^a^	10.59 ± 0.53 ^e^
Day 7	T_1_	7.67 ± 0.38 ^b^	5.37 ± 0.26 ^b^	12.44 ± 0.62 ^d^
	T_2_	7.65 ± 0.38 ^b^	5.35 ± 0.26 ^b^	12.43 ± 0.62 ^d^
	T_3_	7.63 ± 0.38 ^b^	5.32 ± 0.26 ^b^	12.41 ± 0.62 ^d^
	T_4_	7.61 ± 0.38 ^b^	5.29 ± 0.26 ^b^	12.39 ± 0.62 ^d^
	T_5_	7.58 ± 0.37 ^b^	5.25 ± 0.26 ^b^	12.35 ± 0.62 ^d^
Day 14	T_1_	6.12 ± 0.30 ^c^	5.45 ± 0.27 ^b^	13.69 ± 0.68 ^c^
	T_2_	6.11 ± 0.30 ^c^	5.43 ± 0.27 ^b^	13.65 ± 0.68 ^c^
	T_3_	6.11 ± 0.30 ^c^	5.41 ± 0.27 ^b^	13.63 ± 0.68 ^c^
	T_4_	6.09 ± 0.30 ^c^	5.39 ± 0.26 ^b^	13.61 ± 0.68 ^c^
	T_5_	6.09 ± 0.30 ^c^	5.35 ± 0.26 ^b^	13.58 ± 0.68 ^c^
Day 21	T_1_	5.96 ± 0.29 ^d^	4.81 ± 0.24 ^c^	14.65 ± 0.73 ^b^
	T_2_	5.91 ± 0.29 ^d^	4.79 ± 0.23 ^c^	14.63 ± 0.73 ^b^
	T_3_	5.92 ± 0.29 ^d^	4.75 ± 0.23 ^c^	14.61 ± 0.73 ^b^
	T_4_	5.88 ± 0.29 ^d^	4.73 ± 0.23 ^c^	14.59 ± 0.73 ^b^
	T_5_	5.45 ± 0.27 ^d^	4.71 ± 0.23 ^c^	14.55 ± 0.73 ^b^
Day 28	T_1_	5.43 ± 0.27 ^d^	4.45 ± 0.22 ^c^	15.45 ± 0.77 ^a^
	T_2_	5.41 ± 0.27 ^d^	4.43 ± 0.22 ^c^	15.43 ± 0.77 ^a^
	T_3_	5.37 ± 0.27 ^d^	4.41 ± 0.22 ^c^	15.41 ± 0.77 ^a^
	T_4_	5.35 ± 0.26 ^d^	4.38 ± 0.21 ^c^	15.39 ± 0.77 ^a^
	T_5_	5.33 ± 0.26 ^d^	4.35 ± 0.21 ^c^	15.35 ± 0.77 ^a^

Note: Values with different letters in the same column are significantly different at *p* ≤ 0.05. SD, standard deviation.

## Data Availability

Not applicable.
